# Administration of Micronized Caffeine Using a Novel Oral Delivery Film Results in Rapid Absorption and Electroencephalogram Suppression

**DOI:** 10.3389/fphar.2019.00983

**Published:** 2019-09-10

**Authors:** Rochelle M. Hines, Matthew Khumnark, Ben Macphail, Dustin J. Hines

**Affiliations:** ^1^Department of Psychology, University of Nevada Las Vegas, Las Vegas, NV, United States; ^2^Rapid Dose Therapeutics, Burlington, ON, Canada.

**Keywords:** transmucosal, buccal, bioavability, therapeutic delivery, caffeine (1, 3, 7-trimethyl-1h-purine-2)

## Abstract

Route of administration is well-known to impact factors ranging from absorption and distribution, up through the subjective effects of active ingredients. Different routes of administration confer specific advantages, such as more rapid absorption resulting from intravenous injection, or increased convenience with oral administration, but a combination of both rapid and convenient delivery is highly desirable. QuickStrip™ was designed as a rapidly dissolving thin film matrix that contains active ingredients, which may be promising for rapid and convenient delivery *via* the oral mucosa. To assess the delivery of QuickStrip™, we administered the well-characterized active ingredient caffeine to mice and compared QuickStrip™ to standard oral gavage delivery at an equivalent dose of 20 mg kg^-1^. Using HPLC assessment of serum concentrations of caffeine, we found that QuickStrip™ delivery resulted in higher serum levels of caffeine at 1, 10, and 30 min following administration compared to gavage. QuickStrip™ also produced greater bioavailability compared to gavage, as demonstrated by area under the curve analysis. Caffeine delivered by QuickStrip™ produced robust behavioral activation of locomotion, consistent with gavage caffeine. Electroencephalographic (EEG) assessment of central nervous system effects demonstrated that both gavage and QuickStrip™ caffeine produced suppression of delta and theta, consistent with prior literature on the effects of caffeine. In addition, QuickStrip™ produced a more rapid onset of EEG suppression, supporting the more rapid absorption demonstrated in the serum studies. Collectively, these studies suggest that QuickStrip™ may provide a balance between convenience and rapid onset, offering new options for delivery of therapeutics.

## Introduction

Route of administration is well-known to dramatically affect the absorption, distribution, and metabolism, as well as the physiological and behavioral responses to therapeutics. Delivery of significant concentrations of active medicinal ingredients to a target tissue is often a substantial barrier to therapeutic efficacy and hampers the development of novel therapeutics. Drug delivery *via* intravenous (i.v.) injection is known to permit relatively rapid access to the nervous system, allowing onset of therapeutic effects to also be rapid. However, i.v. injection is limited in clinical application of therapeutics by ease of use and risks of injury and infection. Administration *via* injection more generally is known to be frightening and painful and can negatively impact patient compliance (Spain et al., 2016). Oral administration is convenient, yet it typically has a very slow onset of therapeutic effects, and drug potency can be lost because of the action of the digestive system and/or first pass metabolism (Patel et al., 2011; Satheesh Madhav et al., 2012). Novel orodispersible tablets have helped to circumvent some of these issues; however, patient convenience and compliance could still be improved by advances in oral delivery (Reiner et al., 2010). In particular, oral tablet delivery can be challenging for children, the elderly, and severely ill populations, such as those suffering from mucositis following chemotherapy treatment (Reiner et al., 2010).

Absorption of drugs across the buccal or sublingual mucosae is an attractive alternative to standard oral administration because it can bypass first pass metabolism in the liver as well as degradation in the digestive tract (Shojaei, 1998; Patel et al., 2011; Satheesh Madhav et al., 2012). The buccal and sublingual mucosae both receive abundant blood supply and have a relatively high permeability, allowing drugs to enter and act rapidly (Pather et al., 2008; Patel et al., 2011; Satheesh Madhav et al., 2012). Important practical applications for administration *via* the oral mucosa include emergency situations where rapid administration by nonskilled personnel could be life saving, in unconscious patients where swallowing is impaired, and in young children, the elderly, and the severely ill. Administration *via* the oral mucosa also does not require water to administer as an oral tablet would (Reiner et al., 2010). Despite the rationale to develop these methods of delivery, some challenges are still present in buccal or sublingual administration, such as the characteristics of the oral cavity, including pH, enzymatic activity, and permeability of the mucosa (Rathbone et al., 1994; Patel et al., 2011). Improvements to the buccal or sublingual delivery substrate could potentially further speed absorption and distribution of active ingredients. To advance the feasibility and efficacy of drug delivery *via* the oral mucosa, we have examined a novel delivery device consisting of micronized active ingredients incorporated into a therapeutic thin film matrix known as QuickStrip™. This new delivery device has the potential to capitalize on the convenience of oral administration yet allow rapid absorption into the bloodstream and rapid onset of therapeutic effects.

In the present study, we examined the efficacy of therapeutics delivered *via* QuickStrip™ compared to standard oral administration using a well-known active ingredient, caffeine. Caffeine was administered at 20 mg kg^-1^ *via* oral gavage or QuickStrip™ delivery to wild-type C57Bl6J mice, followed by HPLC analysis of blood serum caffeine concentrations at multiple time points, as well as behavioral and electroencephalographic (EEG) assessment of physiological effects. We found that caffeine delivered *via* QuickStrip™ resulted in significantly higher concentrations of caffeine in the serum compared to standard oral gavage as quickly as 1 min following administration and resulted in substantially larger area under the curve (AUC). We next examined the behavioral response to QuickStrip™ caffeine and observed the well-established behavioral response of motor activation. In the last set of experiments, we used EEG as a biomarker for access to the central nervous system and found that caffeine delivery *via* QuickStrip™ produced a very rapid alteration in EEG activity patterns compared to standard oral gavage.

## Methods

**Animals.** Animals were cared for according to the NIH Guide for the Care and Use of Laboratory Animals (National Research Council (US) Committee for the Update of the Guide for the Care and Use of Laboratory Animals, 2011), and protocols were approved by the Institutional Animal Care and Use Committee (IACUC) of the University of Nevada Las Vegas. Animals were group housed on a 12:12-h light-dark schedule in a humidity- and temperature-controlled room.

**Caffeine Administration.** Caffeine was prepared as a 2 mg ml^-1^ solution in deionized distilled water or as the active ingredient micronized within the QuickStrip™ film substrate to deliver 20 mg kg^-1^ caffeine. Mice were weighed the morning of the experiment to calculate dose. Oral gavage was performed using a specialized gavage needle (Sigma-Aldrich) affixed to a 1-ml syringe (Sigma-Aldrich). Briefly, animals were gently restrained, and the gavage was carefully passed through the mouth, down to the depth of the last rib (∼stomach). QuickStrip™ caffeine thin films were obtained from Rapid Dose Therapeutics Corp. The area of the strip was calculated to achieve 20 mg kg^-1^ based on the weight of the mouse. To administer the strip, animals were gently restrained, and the strip was placed against the buccal tissue and held in place with forceps until softened. As a vehicle control, we also administered deionized distilled water *via* gavage.

**Cardiac Puncture and Serum Preparation.** Animals were deeply anesthetized with isoflurane and restrained. Cardiac puncture was performed at specific time points (1, 5, 10, 30, and 60 min; n = 5 per time point, per administration group) following administration by trained personnel by inserting a needle (Sarstedt) affixed with a serum vacutainer (Sarstedt) directly into the heart. Once sufficient sample was obtained, mice were sacrificed. Whole blood was allowed to clot at room temperature for 30 min and then spun at 5,000 x g to obtain serum samples. Serum was pipetted off and stored at -80°C until HPLC analysis (Cyprotex). Serum concentrations of caffeine were compared at each time point using t-test, and graphs were plotted as box plots to display the distribution of the data (SigmaPlot). To examine AUC, scatterplots were generated, and AUC analysis was run using SigmaPlot.

**Behavior.** The open-field apparatus was based on that used in the EMPReSS resource and was 44 cm × 44 cm (Brown et al., 2005). Mice were habituated to the testing room for 1 h before behavioral analysis. Animals were administered either 20 mg kg^-1^ caffeine *via* gavage (n = 8) or QuickStrip™ film (n = 6) or gavage with a vehicle control solution (n = 7). Immediately after administration, animals were placed into the open field for 60 min of free exploration. In the prehabituated studies, mice were first habituated to the open-field arena for 60 min, followed by administration of either 20 mg kg^-1^ caffeine *via* gavage (n = 5) or QuickStrip™ film (n = 4). Immediately after administration, animals were placed into the open field for an additional 60 min of free exploration. Open-field behavior of mice was assessed using the ANY-maze tracking analysis on video recordings taken from above the open field. Paths were plotted from x, y coordinates, and heat maps were generated using MatLab. Speed and distance measures were compared using ANOVA (one way, average distance and average speed; two-way repeated measures, distance traveled over time), and graphs were plotted as mean ± standard error (SigmaPlot).

**Electroencephalography.** EEG and electromyography (EMG) electrodes were implanted under isoflurane anesthesia. Two channels of EEG were recorded bilaterally from the frontal cortex, and ground is supplied by placement in the caudal parietal area. A total of 11 mice were used in the EEG studies, divided randomly into gavage (n = 4) or QuickStrip™ (n = 7) groups. EEG data before administration were pooled to provide baseline (n = 11), allowing the same animals to be used as their own control. After a minimum of 7 days of postoperative recovery, EEG activity was recorded at a sampling rate of 1,000 Hz using the Pinnacle system for mouse during the dark phase of the cycle. Mice were first acclimatized to the recording chamber and preamplifier for 1 h, then recorded for 1 h of baseline. During baseline recordings, sleep was suppressed or interrupted by introduction of novel objects as necessary. After 1 h of baseline, mice were administered 20 mg kg^-1^ caffeine *via* either gavage or QuickStrip™. Recordings proceeded for 1 h following administration. Results were quantified using SleepSign and MatLab. Transformed data were parsed into spectral frequency divisions (Hines et al., 2018) according to the following limits: δ-0.5–4.0 Hz, θ-4.5–8 Hz, α-8.5–13 Hz, β-13.5–30, γ-30.5–100 Hz (Drinkenburg et al., 2015). Spectral analysis compared 1 h of baseline to 1 h postadministration. Time to suppression analysis was performed by measuring the time from drug administration to first suppressed peak, as marked by a change in power of greater than 2 standard deviations from the mean baseline. Results were analyzed using ANOVA (one way, spectral band normalized power and time to suppression; two-way repeated measures, FFT spectral analysis), and graphs were plotted as mean ± standard error (SigmaPlot).

## Results

### Rapid Delivery of Caffeine With Quickstrip™ Compared to Standard Oral Gavage

**Figure 1:**
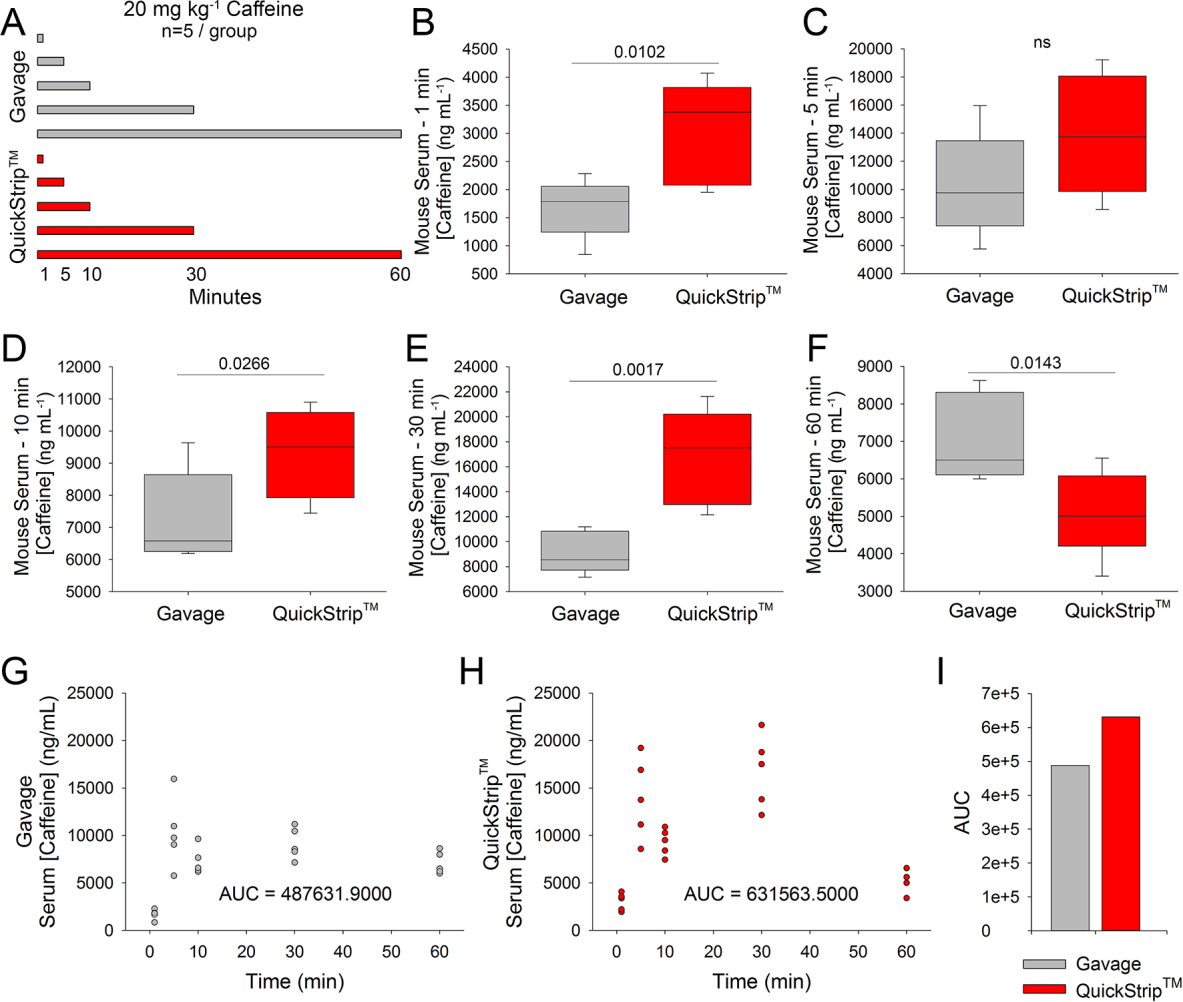
HPLC analysis of serum caffeine concentration following administration of 20 mg kg^-1^ caffeine *via* QuickStrip™ or gavage. **(A)** Visual representation of the experimental design, showing two administration methods (QuickStrip™ or gavage) with five time points where serum was extracted. **(B)** Box plot comparing caffeine concentration in the serum at 1 min following gavage or QuickStrip™ administration. **(C)** Box plot comparing caffeine concentration in the serum at 5 min following gavage or QuickStrip™ administration (ns, not significant; p = 0.0950). **(D)** Box plot comparing caffeine concentration in the serum at 10 min following gavage or QuickStrip™ administration. **(E)** Box plot comparing caffeine concentration in the serum at 30 min following gavage or QuickStrip™ administration. **(F)** Box plot comparing caffeine concentration in the serum at 60 min following gavage or QuickStrip™ administration. p values were calculated using t-test to compare means. **(G**, **H)** Scatter plots showing the concentration of caffeine in serum across all time points for gavage and QuickStrip™, respectively. **(I)** Comparison of the AUC between gavage and QuickStrip™. AUC was calculated using SigmaPlot for each scatterplot.

Sublingual delivery *via* the QuickStrip™ film substrate has the potential to more rapidly deliver active ingredients compared to standard oral administration. To quantitatively assess this, we administered 20 mg kg^-1^ caffeine to C57Bl6 mice *via* standard oral gavage or QuickStrip™ and performed cardiac puncture to obtain blood samples at 1, 5, 10, 30, and 60 min following administration (**Figure 1A**). Serum samples were prepared from whole blood and analyzed by HPLC to obtain caffeine concentration values. We compared caffeine concentration values obtained with gavage and QuickStrip™ caffeine and found that at 1 min, significantly greater caffeine concentration (3,034.600 ± 408.372 ng/ml) was detected in the serum when caffeine was delivered to mice *via* QuickStrip™ compared to gavage (1,677.440 ± 234.864 ng ml^-1^; p = 0.0102; **Figure 1B**). We also found that QuickStrip™ caffeine resulted in higher serum concentrations at 10 (9,304.200 ± 622.393 ng ml^-1^) and 30 (16,776.000 ± 1,708.366 ng ml^-1^) min postadministration in comparison to gavage (7,275.000 ± 643.907 ng ml^-1^; p = 0.0266; 9,129.400 ± 738.533 ng ml^-1^; p = 0.00170; **Figures 1D, E**). No significant difference was detected at 5 min between gavage and QuickStrip™ caffeine (13,919.400 ± 1,913.372 ng ml^-1^; 10,294.000 ± 1,657.015 ng ml^-1^; p = 0.0950; **Figure 1C**), and by 60 min, the serum concentration produced by QuickStrip™ (5,115.800 ± 512.726 ng ml^-1^) fell below that produced by gavage (7,068.200 ± 523.130 ng ml^-1^; p = 0.0143; **Figure 1F**). These results demonstrate that QuickStrip™ produced more rapid delivery of the active ingredient, caffeine, compared to oral administration.

### Greater Bioavailability With QuickStrip™ Administration of Caffeine

**Figure 2:**
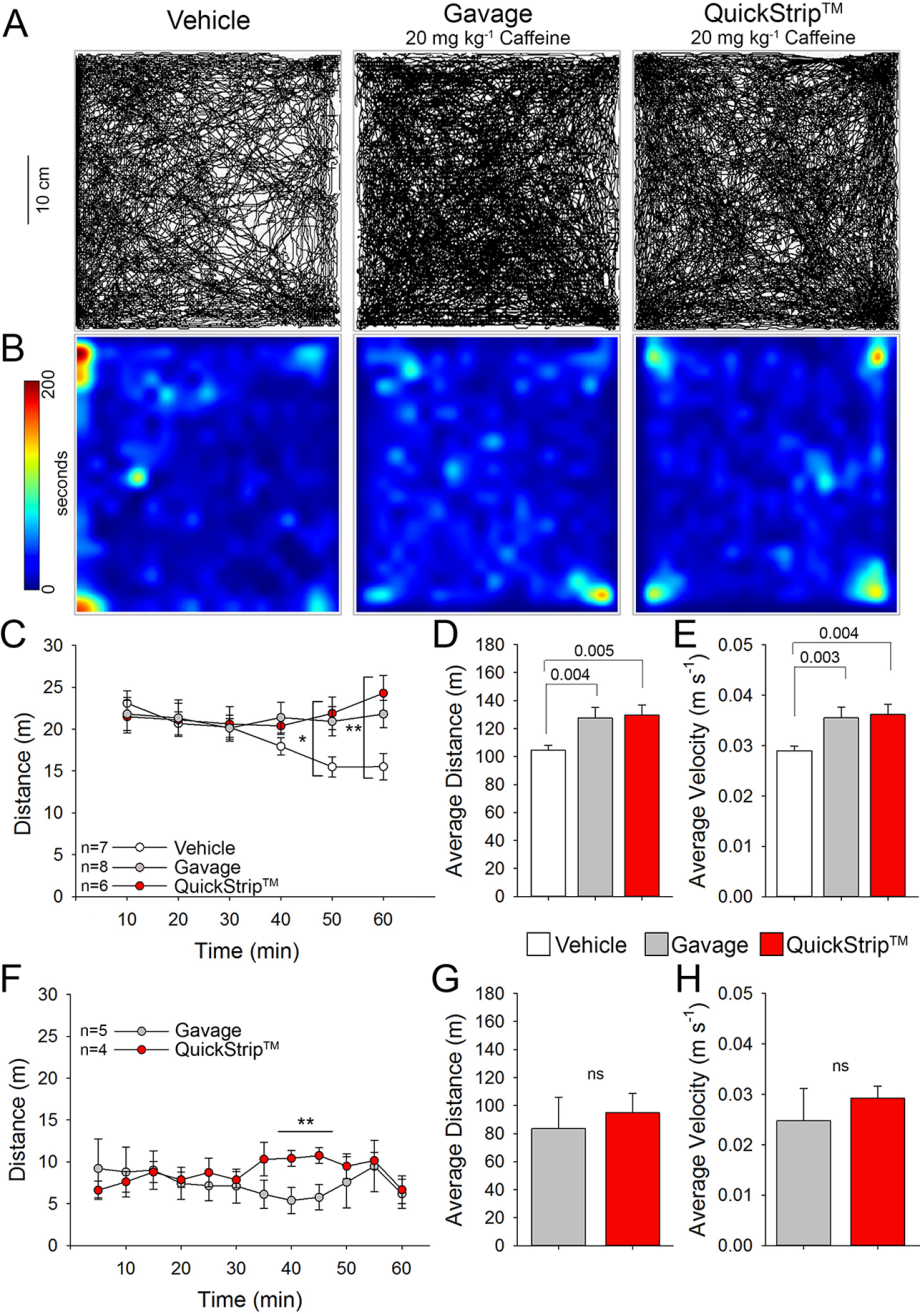
Stimulation of locomotion in the open-field test following administration of 20 mgkg^-1^ of caffeine *via* either QuickStrip™ or gavage. **(A)** Representative paths of animals treated with vehicle (distilled H_2_O gavage, left), gavage (middle), and QuickStrip™ (right). **(B)** Representative heat maps showing animal dwell time in the open-field apparatus with warmer colors indicating longer dwell time. **(C)** A plot of average distance traveled over time after administration of 20 mg kg^-1^ caffeine *via* gavage or QuickStrip™ compared to vehicle control. (* vehicle vs gavage p = 0.044; vehicle vs QuickStrip™ p = 0.038; **vehicle vs gavage p = 0.017; vehicle vs QuickStripTM p = 0.002). **(D)** Bar plot of total distance traveled during the 60 min testing period. **(E)** Bar plot describing the average velocity throughout the 60 min testing period. **(F)** A plot of average distance traveled over time when mice were habituated to the open field followed by administration of 20 mg kg^-1^ caffeine *via* gavage or QuickStrip™ (**40 min p = 0.016; 45 min p = 0.020).. **(G)** Bar plot of total distance traveled during the 60 min testing period after mice were habituated to the open field. (ns p = 0.277). **(H)** Bar plot describing the average velocity throughout the 60 min testing period after mice were habituated to the open field. (ns p = 0.288). Mean ± standard error, p values were calculated using repeated measures **(C**, **F)** or one-way ANOVA **(D**, **E**, **G**, **H)** in SigmaPlot.

In addition to rapid delivery, the QuickStrip™ substrate has the potential to deliver greater overall serum concentrations of active ingredient compared to standard oral administration. To assess the overall bioavailability of caffeine, we analyzed the HPLC results from both oral gavage and QuickStrip™ for the AUC. Serum results across the time course were plotted for both gavage and QuickStrip™ caffeine (**Figure 1G,H**). Plots reveal a second rise in serum concentration at 30 min post QuickStrip™ administration (**Figure 2H**). AUC analysis of gavage administration resulted in a value of 487,631.9, whereas AUC analysis of QuickStrip™ resulted in a value of 631,563.5 (**Figure 1I**), resulting in a difference of 1,439,931.6 between the calculated AUCs. These results demonstrate that QuickStrip™ delivery of caffeine produced greater bioavailability than standard oral gavage administration.

### QuickStrip™ Caffeine Produces Reliable Increases in Behavioral Arousal

To confirm the effectiveness of QuickStrip™ caffeine delivery using a functional outcome in mice, we assessed stimulatory effects on locomotion in the open field. Moderate doses of caffeine are well-known to produce locomotor stimulation in mice (El Yacoubi et al., 2000; Karcz-Kubicha et al., 2003), as well as in humans (Swerdlow et al., 1986; Orrú et al., 2013). We selected a dose of 20 mg kg-1, which has been used in mice to produce robust locomotor stimulation and is below the high doses of caffeine that cause locomotor suppression (100 mg kg^-1^; El Yacoubi et al., 2000). Naive C57Bl6 mice were administered 20 mg kg^-1^ caffeine *via* either standard oral gavage or QuickStrip™ (or vehicle control gavage) and placed in the open-field arena. Mice were allowed to move freely in the open field for 1 h. The representative paths and heat maps for gavage and QuickStrip™ caffeine delivery show a high level of locomotor activity in comparison to the vehicle control (**Figures 2A, B**). Analysis of distance traveled over time in the open field demonstrated that QuickStrip™ caffeine (least square mean = 21.632 m) produces robust locomotor activation that lasts the duration of the task, similar to an equal dose of gavage caffeine (least square mean = 21.234 m; **Figure 2C**). Both QuickStrip™ and gavage caffeine-administered mice were more active than vehicle control mice (least square mean = 18.833 m), with significantly greater distance traveled at both 50 (QuickStrip™ versus vehicle, p = 0.038; gavage versus vehicle, p = 0.044) and 60 (QuickStrip™ versus vehicle, p = 0.002; gavage versus vehicle, p = 0.017) min of the open-field test. Assessment of the average cumulative distance (**Figure 2D**) reveals that both gavage (127.401 ± 5.300 m) and QuickStrip™ (129.793 ± 4.845 m) caffeine produce stimulatory effects on locomotion (p = 0.004; p = 0.005). Additional assessment of average speed (**Figure 2E**) also shows that both gavage (0.0355 ± 0.00145 m s^-1^) and QuickStrip™ (0.0362 ± 0.00136 m s^-1^) caffeine produce stimulatory effects on locomotion (p = 0.003; p = 0.004).

In addition to administration to naive mice in the open field, we also compared the effects of QuickStrip™ and gavage caffeine after prior exposure to the open-field environment. When first placed in a novel environment, animals experience heightened activity levels that decline over time spent exploring the environment, an effect referred to as habituation (Deacon, 2006). Because of the effect of the novel environment, all groups of mice are highly mobile and travel a relatively large distance. To further compare the routes of administration, we eliminated the effect of the novel environment by first habituating naive mice to the open field before administration of caffeine *via* QuickStrip™ or gavage. Analysis of distance traveled over time in the open field following habituation and administration demonstrated that QuickStrip™ caffeine (least square mean = 8.773 m) produces a response similar to an equal dose of gavage caffeine (least square mean = 9.273 m; **Figure 2F**). There was a significant interaction between caffeine route of administration and time, with QuickStrip™ producing an elevated effect after 35 min. This second phase of enhanced behavioral arousal may be reflective of the second rise in serum concentration observed following QuickStrip™ administration of caffeine. The average cumulative distance traveled (**Figure 2G**; QuickStrip™: 94.856 ± 8.963 m; gavage: 83.635 ± 14.921 m) and average speed (**Figure 2H**; QuickStrip™: 0.0292 ± 0.00245 m s^-1^; gavage: 0.0248 ± 0.00641 m s^-1^) did not differ between QuickStrip™ and gavage caffeine. The behavioral results demonstrated that QuickStrip™ caffeine produces robust locomotor activation similar to that found with gavage oral administration, which is consistent with the expected effects of moderate doses of caffeine.

### More Rapid Effects on the Central Nervous System With QuickStrip™ Caffeine as Assessed by Electroencephalography

**Figure 3:**
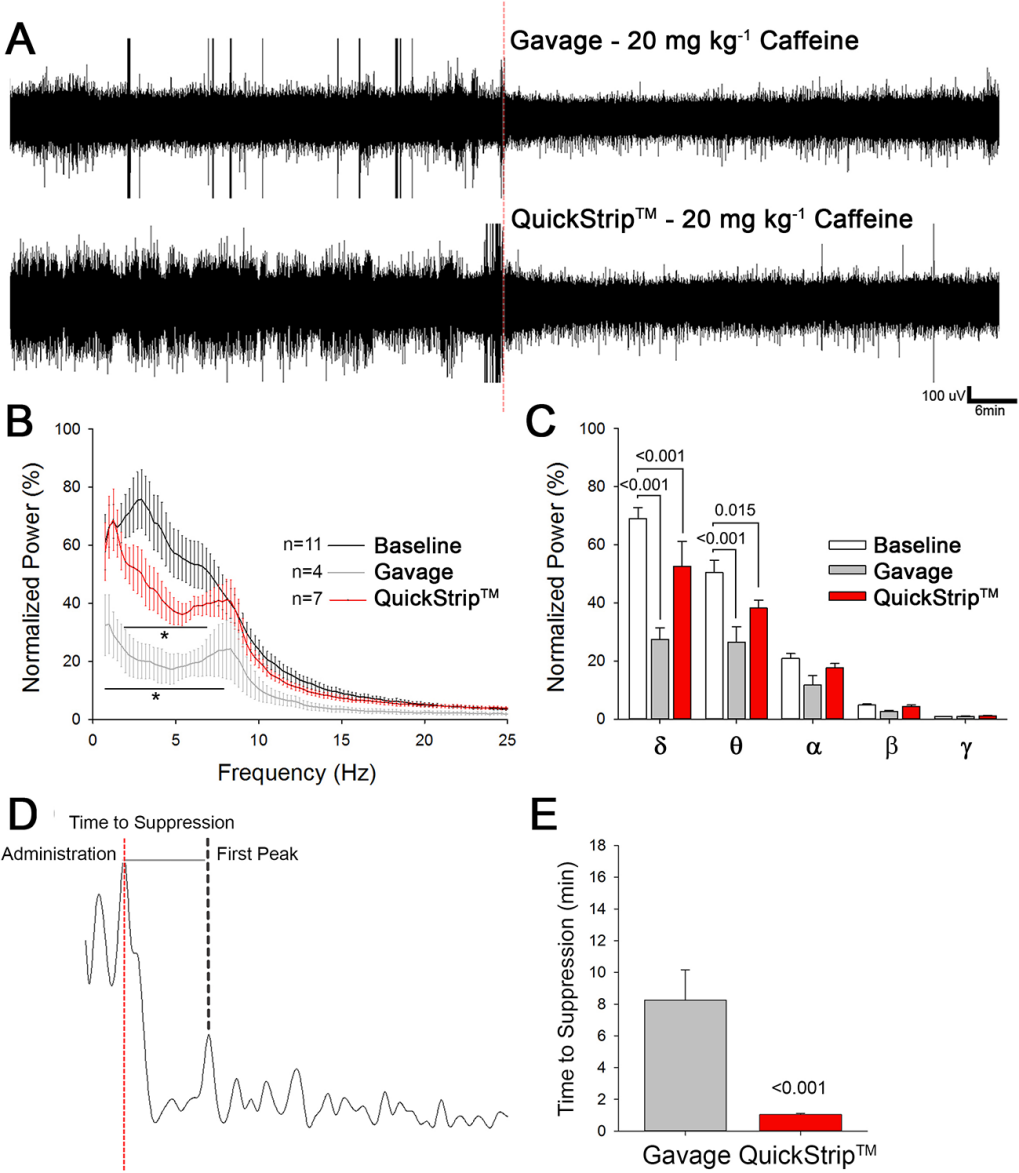
Electroencephalographic analysis comparing the administration of 20 mg kg^-1^ caffeine *via* Quickstrip™ and gavage. **(A)** Two-hour raw traces, with the first hour as awake baseline and second with administration of caffeine using gavage or Quickstrip™, marked at the red dotted line. **(B)** Cumulative Fast Fourier Transform (cFFT) analysis of EEG data, normalized to baseline. **(C)** Bar graph showing mean normalized power, separated by frequency band δ-0.5–4.0 Hz, θ-4.5–8 Hz, α-8.5–13 Hz, β-13.5–30, γ-30.5–100 Hz. **(D)** Representative 10-s trace of delta power observed after administration *via* either gavage or Quickstrip™ (time of administration marked by the dotted red line). **(E)** Bar graph comparing average time for observed delta suppression to occur. Mean ± standard error, p values were calculated using repeated measures **(B**, **C)** or one-way ANOVA **(E)** in SigmaPlot.

To sensitively examine the onset of central nervous system effects following either gavage or QuickStrip™ caffeine, we used EEG. EEG is well-known to provide a readout of central nervous system activity, and specific changes in the EEG follow administration of different classes of drugs. Caffeine has previously been shown to suppress low-frequency oscillations, particularly delta (띤δ) band activity (Landolt et al., 1995; Landolt et al., 2004; Paterson et al., 2009). The EEG suppression induced by caffeine is evident from the raw traces (**Figure 3A**). Fast-Fourier transform analysis reveals potent suppression in low-frequency oscillations resulting from both gavage and QuickStrip™ caffeine delivery (**Figure 3B**). To analyze in more detail, we parsed the EEG into spectral frequency bands δ-0.5–4.0 Hz, θ-4.5–8 Hz, α-8.5–13 Hz, β-13.5–30, γ-30.5–100 Hz (Drinkenburg et al., 2015) and compared spectral frequencies between 1 h of baseline and 1 h following either gavage or QuickStrip™ caffeine delivery. We found that δ power was significantly suppressed by caffeine as delivered by gavage (difference of means = 41.497; p < 0.001) and QuickStrip™ (difference of means = 16.336; p < 0.001) compared to baseline (**Figure 3C**). θ band power was also significantly suppressed by both gavage (difference of means = 23.982; p < 0.001) and QuickStrip™ (difference of means = 12.223; p = 0.015) caffeine. To assess how rapidly the suppression took place, we measured the time from drug administration to first suppressed peak, as marked by a change in power of greater than 2 standard deviations from the mean baseline (**Figure 3D**). We found that caffeine delivery by QuickStrip™ produced suppression of the EEG more rapidly than gavage delivery, with average onsets ofs suppression of 1.038 min and 8.249 min, respectively (p < 0.001; **Figure 3E**). These data demonstrated that QuickStrip™ caffeine produced the expected effects of low-frequency EEG suppression, with a more rapid onset compared to gavage.

## Discussion

In these studies, we used HPLC analysis of mouse serum to demonstrate that QuickStrip™ delivered the active ingredient caffeine more rapidly and with greater bioavailability as compared to gavage administration. We have also shown that QuickStrip™ caffeine produced the expected behavioral effects of locomotor stimulation, consistent with gavage administration of caffeine. Quantitative EEG studies demonstrated that QuickStrip™ caffeine produced rapid central nervous system effects, suppressing low-frequency EEG more rapidly than gavage. Collectively, these data demonstrated that the QuickStrip™ delivery substrate may be a viable option to balance ease of use with rapid delivery.

Serum data revealed rapid absorption of QuickStrip™ caffeine compared to gavage, with substantial concentrations of caffeine detected within 1 min of QuickStrip™ delivery. The greatest variability is observed within the QuickStrip™ caffeine group at the 5-min time point following administration. This variability could be caused by individual differences in salivation, with high rates of salivation increasing the pH of the oral cavity and potentially influencing absorption (Rathbone et al., 1994; Madhav et al., 2009; Gittings et al., 2015). Also of interest is the second peak in serum caffeine concentration observed at 30 min following QuickStrip™ delivery, again with higher variance. This may have also resulted from saliva flow in the individual subjects, which can lead to swallowing before absorption *via* the oral mucosa is complete (Patel et al., 2011). The second peak may allow for longer duration of effect and may be a desirable component of QuickStrip™. Analysis of AUC demonstrates increased bioavailability of caffeine when delivered by QuickStrip™ as compared to gavage. Collectively, the analysis of HPLC serum caffeine studies reveals that QuickStrip™ provides more rapid and greater overall absorption of an active ingredient into the blood supply.

QuickStrip™ and gavage caffeine delivery both produced behavioral activation in the mice, as demonstrated by increased locomotion in the open field. Given the rapid absorption and greater bioavailability of QuickStrip™ caffeine, one might have expected more rapid and robust effects on locomotion. However, behavioral assessment in the open field does not allow for examination of rapid onset of locomotor effects because of the high level of activity of all mice when placed in the novel environment of the open field. There may also be a “ceiling” to the level of locomotor activation occurring between 12.5 and 25 mg kg^−1^, as higher doses of caffeine do not produce activation (50 mg kg^−1^) and can produce suppression of locomotor activity (El Yacoubi et al., 2000). Overall, these experiments demonstrate that QuickStrip™ caffeine produced the expected locomotor activation in the open field.

QuickStrip™ caffeine produced rapid suppression of EEG, which mirrored the serum data. Previous studies in humans and in nonhuman animals have demonstrated that caffeine suppresses power particularly in the lower frequencies (Landolt et al., 1995, Landolt et al., 2004; Paterson et al., 2009; Van Dort et al., 2009). The effects of suppressing EEG and increasing locomotor activity are interrelated, stemming from caffeine’s antagonistic action on adenosine A1 and A2 receptors and downstream regulation of acetylcholine release (Van Dort et al., 2009). In particular, it is proposed that A1 receptors within the prefrontal cortex are involved in descending inhibition of wakefulness (Van Dort et al., 2009), and consequently, when A1 receptors are inhibited by caffeine, increased vigilance or arousal results. Arousal from sleep to wake alone is not sufficient to explain the suppression of EEG δ-power following caffeine administration because mice were not allowed to sleep during baseline recordings. Whereas the EEG suppression appears more apparent in the gavage caffeine-treated mice, the relationship between dose and/or serum concentration and EEG effects is not always linear. A higher dose producing a greater serum concentration may lead to a reversal of a lower dose effect, analogous to the behavioral response to caffeine, which at low to moderate doses produce behavioral activation, whereas at high doses, it produces behavioral arrest (El Yacoubi et al., 2000).

Using EEG, we were able to detect robust suppression of the EEG power approximately 1 min following caffeine administration *via* QuickStrip™, indicating that caffeine rapidly entered the nervous system to exert these effects. By comparison, we found that gavage administration resulted in suppression at approximately 8 min. The EEG data reflected the rapid absorption of QuickStrip™ caffeine that was also demonstrated with HPLC assessment of caffeine concentration in the serum. Paired with the serum data, these EEG studies demonstrated that QuickStrip™ caffeine is rapidly absorbed, permitting rapid access to the nervous system. This result is particularly significant, as access to the central nervous system is a key factor in the efficacy of psychoactive therapies. The blood–brain barrier restricts access to the central nervous system, preventing exposure to dangerous substances, but also limiting the efficacy of many compounds. The ability of caffeine delivered *via* QuickStrip™ to be absorbed into the serum and impact brain activity rapidly suggests that this route of administration may offer advantages, particularly for psychoactive compounds.

Caffeine has clinical application in the treatment of headache and is routinely used alone or in combination with other treatments by headache patients (Fried et al., 2017; Lipton et al., 2017). Combinations including caffeine significantly improve the efficacy of analgesic medication monotherapy for the treatment of patients with tension headache or migraine (Diener et al., 2014; Goldstein et al., 2014). Additional benefit has been suggested with rapid delivery, such as with the use of intravenous administration of caffeine (Baratloo et al., 2017); however, for many patients, this route of administration is impractical. QuickStrip™ caffeine may provide convenient yet more rapid delivery than an orally administered analgesic that includes caffeine. Again, being able to rapidly act on the brain is a particular benefit, as the rapid alleviation of symptoms is desirable and limits secondary aspects of headache and migraine from developing. A thin, rapidly dissolving film delivered to the oral mucosa also offers advantages over oral tablets in terms of convenience and may offer improved patient comfort and compliance (Reiner et al., 2010). In summary QuickStrip™ offers both rapid and functional delivery of caffeine, with greater bioavailability and more rapid central nervous system effects compared to standard oral administration. Further development of micronized dispersions of therapeutics for buccal and sublingual administration will advance available options for patients and has the potential to improve health outcomes.

## Data Availability

The datasets generated for this study are available on request to the corresponding author.

## Ethics Statement

Animals were cared for according to the NIH Guide for the Care and Use of Laboratory Animals (National Research Council (US) Committee for the Update of the Guide for the Care and Use of Laboratory Animals, 2011) and protocols were approved by the Institutional Animal Care and Use Committee (IACUC) of the University of Nevada Las Vegas.

## Author Contributions

RH contributed to designing and conducting experiments, preparation of figures and writing of the manuscript. MK. contributed to conducting experiments and analyzing data. BM formulated strips and contributed to the manuscript. DH contributed to designing and conducting experiments, preparation of figures and writing of the manuscript.

## Funding

This work was supported through an industry sponsored research agreement with Rapid Dose Therapeutics. The funder was not involved in the study design, collection, analysis, interpretation of data, the writing of this article or the decision to submit it for publication. Publishing costs were supported by the UNLV College of Liberal Arts.

## Disclaimer

Copyright permission for the use of Quickstrip™ is granted through a master agreement *via* the University of Nevada Las Vegas and also through a letter from the CEO (owner of copyright permission).

## Conflict of Interest Statement

RH and DH act as consultants for Rapid Dose Therapeutics, relationships that are regulated by the University of Nevada Las Vegas.

The remaining authors declare that the research was conducted in the absence of any commercial or financial relationships that could be construed as a potential conflict of interest.

## References

[B1] Baratloo A. Mirbaha S. Delavar Kasmaei H. Payandemehr P. Elmaraezy A. Negida A. (2017). Intravenous caffeine citrate vs. magnesium sulfate for reducing pain in patients with acute migraine headache; a prospective quasi-experimental study. Korean J. Pain 30, 176–182. 10.3344/kjp.2017.30.3.176 28757917 PMC5532524

[B2] Brown S. D. M. Chambon P. de Angelis M. H., and Eumorphia Consortium (2005). EMPReSS: standardized phenotype screens for functional annotation of the mouse genome. Nat. Genet. 37, 1155. 10.1038/ng1105-1155 16254554

[B3] Deacon R. M. (2006). Housing, husbandry and handling of rodents for behavioral experiments. Nat. Protoc. 1 (2), 936–946. 10.1038/nprot.2006.120 17406327

[B4] Diener H.-C. Gold M. Hagen M. (2014). Use of a fixed combination of acetylsalicylic acid, acetaminophen and caffeine compared with acetaminophen alone in episodic tension-type headache: meta-analysis of four randomized, double-blind, placebo-controlled, crossover studies. J. Headache Pain 15, 76. 10.1186/1129-2377-15-76 25406671 PMC4256978

[B5] Drinkenburg W. H. I. M. Ruigt G. S. F. Ahnaou A. (2015). ). Pharmaco-EEG studies in animals: an overview of contemporary translational applications. Neuropsychobiology 72, 151–164. 101159/000442210. 26901596 10.1159/000442210

[B6] El Yacoubi M. Ledent C. Ménard J. F. Parmentier M. Costentin J. Vaugeois J. M. (2000). The stimulant effects of caffeine on locomotor behaviour in mice are mediated through its blockade of adenosine A(2A) receptors. Br. J. Pharmacol. 129, 1465–1473. 10.1038/sj.bjp.0703170 10742303 PMC1571962

[B7] Fried N. T. Elliott M. B. Oshinsky M. L. (2017). The role of adenosine signaling in headache: a review. Brain Sci. 7 (3), E30. 10.3390/brainsci7030030. 28335379 PMC5366829

[B8] Gittings S. Turnbull N. Henry B. Roberts C. J. Gershkovich P. (2015). Characterisation of human saliva as a platform for oral dissolution medium development. Eur. J. Pharm. Biopharm. 91, 16–24. 10.1016/j.ejpb.2015.01.007 25603197

[B9] Goldstein J. Hagen M. Gold M. (2014). Results of a multicenter, double-blind, randomized, parallel-group, placebo-controlled, single-dose study comparing the fixed combination of acetaminophen, acetylsalicylic acid, and caffeine with ibuprofen for acute treatment of patients with severe migraine. Cephalalgia 34, 1070–1078. 10.1177/0333102414530527 24733408

[B10] Hines R. M. Maric H. M. Hines D. J. Modgil A. Panzanelli P. Nakamura Y. (2018). Developmental seizures and mortality result from reducing GABA(A) receptorα2-subunit interaction with collybistin. Nat. Commun. 9 (1), 3130. 10.1038/s41467-018-05481-1 30087324 PMC6081406

[B11] Karcz-Kubicha M. Antoniou K. Terasmaa A. Quarta D. Solinas M. Justinova Z. (2003). Involvement of adenosine A1 and A2A receptors in the motor effects of caffeine after its acute and chronic administration. Neuropsychopharmacology 28, 1281–1291. 10.1038/sj.npp.1300167 12700682

[B12] Landolt H. P. Dijk D. J. Gaus S. E. Borbély A. A. (1995). Caffeine reduces low-frequency delta activity in the human sleep EEG. Neuropsychopharmacology 12, 229–238. 10.1016/0893-133X(94)00079-F 7612156

[B13] Landolt H.-P. Rétey J. V. Tönz K. Gottselig J. M. Khatami R. Buckelmüller I. (2004). Caffeine attenuates waking and sleep electroencephalographic markers of sleep homeostasis in humans. Neuropsychopharmacology 29, 1933–1939. 10.1038/sj.npp.1300526 15257305

[B14] Lipton R. B. Diener H.-C. Robbins M. S. Garas S. Y. Patel K. (2017). Caffeine in the management of patients with headache. J. Headache Pain 18, 107. 10.1186/s10194-017-0806-2 29067618 PMC5655397

[B15] Madhav N. V. S. Shakya A. K. Shakya P. Singh K. (2009). Orotransmucosal drug delivery systems: a review. J. Controlled Release 140, 2–11. 10.1016/j.jconrel.2009.07.016 19665039

[B16] National Research Council (US) Committee for the Update of the Guide for the Care and Use of Laboratory Animals (2011). Guide for the Care and Use of Laboratory Animals. 8th Washington (DC): National Academies Press (US) Available at: http://www.ncbi.nlm.nih.gov/books/NBK54050/ [Accessed March 28, 2019].

[B17] Orrú M. Guitart X. Karcz-Kubicha M. Solinas M. Justinova Z. Barodia S. K. (2013). Psychostimulant pharmacological profile of paraxanthine, the main metabolite of caffeine in humans. Neuropharmacology 67, 476–484. 10.1016/j.neuropharm.2012.11.029 23261866 PMC3562388

[B18] Patel V. F. Liu F. Brown M. B. (2011). Advances in oral transmucosal drug delivery. J. Controlled Release 153, 106–116. 10.1016/j.jconrel.2011.01.027 21300115

[B19] Paterson L. M. Wilson S. J. Nutt D. J. Hutson P. H. Ivarsson M. (2009). Characterisation of the effects of caffeine on sleep in the rat: a potential model of sleep disruption. J. Psychopharmacol. (Oxford) 23, 475–486. 10.1177/0269881109104846 19395429

[B20] Pather S. I. Rathbone M. J. Senel S. (2008). Current status and the future of buccal drug delivery systems. Expert Opin. Drug Deliv. 5, 531–542. 10.1517/17425247.5.5.531 18491980

[B21] Rathbone M. J. Drummond B. K. Tucker I. G. (1994). The oral cavity as a site for systemic drug delivery. Adv. Drug Delivery Rev. 13, 1–22. 10.1016/0169-409X(94)90024-8

[B22] Reiner V. Giarratana N. Monti N. C. Breitenbach A. Klaffenbach P. (2010). Rapidfilm: aninnovativepharmaceuticalformdesigned to improvepatient compliance. Int. J. Pharm. 393 (1-2), 55–60. 10.1016/j.ijpharm.2010.03.055 20363308

[B23] Satheesh Madhav N. V. Semwal R. Semwal D. K. Semwal R. B. (2012). Recent trends in oral transmucosal drug delivery systems: an emphasis on the soft palatal route. Expert Opin. Drug Deliv. 9, 629–647. 10.1517/17425247.2012.679260 22512535

[B24] Shojaei A. H. (1998). Buccal mucosa as a route for systemic drug delivery: a review. J. Pharm. Pharm. Sci. 1, 15–30. 10942969

[B25] Spain C. V. Wright J. J. Hahn R. M. Wivel A. Martin A. A. (2016). Self-reported barriers to adherence and persistence to treatment with injectable medications for type 2 diabetes. Clin. Ther. 38, 1653–1664.e1. 10.1016/j.clinthera.2016.05.009 27364806

[B26] Swerdlow N. R. Vaccarino F. J. Amalric M. Koob G. F. (1986). The neural substrates for the motor-activating properties of psychostimulants: a review of recent findings. Pharmacol. Biochem. Behav. 25, 233–248. 10.1016/0091-3057(86)90261-3 2875470

[B27] Van Dort C. J. Baghdoyan H. A. Lydic R. (2009). Adenosine A(1) and A(2A) receptors in mouse prefrontal cortex modulate acetylcholine release and behavioral arousal. J. Neurosci. 29, 871–881. 10.1523/JNEUROSCI.4111-08.2009 19158311 PMC3430130

